# Individual and contextual factors associated with tooth loss among Brazilian older adults

**DOI:** 10.1590/1980-549720260005.supl.1

**Published:** 2026-05-18

**Authors:** Armando Cabral de Lira, Ana Beatriz Rodrigues Moura, Lilian Nadja Silva Brito, Lucas Xavier Bezerra de Menezes, Luciana Leônia Soares Freire, Maria Letícia Barbosa Raymundo, Thayana Maria Navarro Ribeiro de Lima Martins, Rênnis Oliveira da Silva, Yuri Wanderley Cavalcanti, Rafael Aiello Bomfim

**Affiliations:** I Universidade Federal da Paraíba, Graduate Program in Dentistry – João Pessoa (PB), Brazil.; II Universidade Federal de Pernambuco, Graduate Program in Child and Adolescent Health – Recife (PE), Brazil.; III Universidade Federal da Paraíba, Department of Clinical and Social Dentistry – João Pessoa (PB), Brazil.; IV Universidade Federal do Mato Grosso do Sul, Department of Preventive Dentistry – Campo Grande (MS), Brazil.

**Keywords:** Aged, Tooth loss, Health inequalities, Oral health, Socioeconomic factors, Cross-sectional studies

## Abstract

**Objective:**

To assess factors associated with functional dentition, severe tooth loss, and edentulism among Brazilian older adults, investigating individual sociodemographic characteristics and contextual characteristics of municipal oral health services.

**Methods:**

**Results:**

Oral health coverage in Primary Health Care and the presence of CEO and LRPD were not associated with edentulism (p>0.05). Functional dentition was less likely among women (OR 0.66; 95%CI 0.57–0.76) and among residents of municipalities with oral health coverage ≥80% (OR 0.74; 95%CI 0.56–0.97). Higher income and level of education were associated with higher prevalence of functional dentition (p<0.05). Severe tooth loss and edentulism were more frequent among women and older adults with lower income and lower level of education (p<0.05). Severe tooth loss was less prevalent in municipalities with CEO (p<0.05).

**Conclusion:**

Individual socioeconomic variables and contextual characteristics of oral health services were associated with tooth loss among older adults, evidencing the persistence of social inequalities in this outcome.

## INTRODUCTION

Oral diseases are among the most prevalent health conditions in the world and constitute important chronic noncommunicable diseases^1^. Among its outcomes, tooth loss is highlighted, a cumulative consequence of untreated caries and periodontal disease. Throughout the course of life, this burden may result in severe tooth loss, defined by the World Health Organization (WHO) as the presence of less than ten permanent teeth^2^ with repercussions such as pain and suffering, functional impairment, and reduction of quality of life^3^.

In Brazil, according to the National Survey of Oral Health (SB Brasil 2010), there was a mean of 0.4 missing teeth in adolescents (15–19 years), 7.4 in adults (35–44 years), and 25.4 in older adults (65–74 years)^4^. These age differences express cumulative effects of a historical model of oral health care marked by mutilating practices and restrictive preventive actions, insufficient to meet the needs of the population^5^.

Older adults concentrate the greater accumulation of oral disorders and, therefore, constitute a sensitive marker of the effectiveness of public policies over time^6,7^. Severe loss compromises masticatory functions and aesthetic aspects, reduces quality of life and reflects social inequalities, evidencing the capacity — or lack of it — of the public system to offer preventive and rehabilitating care in an equitable way^8^. Ferreira et al.^9^ observed more significant reductions in tooth loss and edentulism among white older people with higher levels of education, showing that advances occurred unevenly among population groups.

Within this context, authors of systematic reviews and international synthesis show that tooth loss in adults and older adults is a global public health issue, with high prevalence and strong social inequalities. Chan et al.^10^, in a systematic review, demonstrated that coronal and root caries remains highly prevalent among older adults in different regions, with high averages of missing teeth and higher burden among socially vulnerable individuals with irregular access to dental services. Moreover, according to international consensus and reviews, care models centered on late, restorative, or surgical interventions are insufficient for the complexity of aging care, reinforcing preventive, longitudinal risk management, person-centered approaches throughout the course of life^11–13^.

In addition to the limitations attributed to the public system, the persistence of the mutilating model should also be understood in the light of the private sector. The commodification of dentistry tends to reconfigure care as a consumer service, with treatments offered as commodities and not based on the patients’ best interest^14,15^. In this scenario, extraction can be imposed as a quick and lower-cost alternative, especially among socially vulnerable populations, reinforcing mutilating practices and inequalities in oral health. According to recent clinical evidence, age, economic restrictions, and limited access to conservative treatments are associated with a higher frequency of surgical procedures over restorative approaches, suggesting that therapeutic decisions are strongly conditioned by structural and organizational determinants of care^16^.

The National Oral Health Policy (*Política Nacional de Saúde Bucal* – PNSB), implemented in 2004 and instituted as a State policy by Law No. 14,572/2023, seeks to expand the offer of promotion and prevention actions and to reduce inequalities in access to dental care^10,17^. The provision of services takes place through the Oral Health Care Network, which articulates different care points. In Primary Health Care (PHC), Oral Health Teams (OHT) and Mobile Dental Units (MDU) provide care; in the specialized care, the Dental Specialty Centers (*Centros de Especialidades Odontológicas* – CEO), Specialized Oral Health Service (*Serviço Especializado em Saúde Bucal* – SESB), and Regional Dental Prosthesis Laboratories (*Laboratórios Regionais de Prótese Dentária* – LRPD) stand out, composing a network focused on the integrality of care^17^.

Although formally structured, the network has been marked by underfunding and undershooting of resources, generating a mismatch between its expansion and the needs of the population. As a consequence, services often provide late responses, focused on pain relief and predominantly interventional practices^18,19^. In this sense, the formulation and implementation of PNSB recognized the role of social determinants of health and prioritized the expansion of the network in more disadvantaged contexts, with worse health indicators^17^.

In the last 20 years, the population aged 65 to 74 years has potentially benefited from the expansion of the provision of public oral health services in the Brazilian Unified Health System (SUS). Given this scenario, it is relevant to investigate the association of individual socioeconomic factors and contextual characteristics of oral health services with the prevalence of tooth loss among older adults. Thus, in the present study, we aim to analyze individual and contextual factors associated with tooth loss in Brazilian older adults.

## METHODS

### Study design

This is a cross-sectional, quantitative, descriptive, and analytical study, based on data from the epidemiological survey SB Brasil 2023. The collection took place between 2022 and 2023, following nationally standardized methodological guidelines. The sample design and methodological procedures were described by Vargas et al.^20^ and Ferreira et al.^9^.

The national survey was approved by a research ethics committee, and all participants signed an Informed Consent Form. The data of this investigation were made available by the Ministry of Health, upon request and signature of an Instrument of Commitment by the researchers.

### Sample

The sample consisted of older adults aged 65–74 years (n=9,745) who answered the individual questionnaire of SB Brasil 2023. Completeness analysis was performed, and no significant differences were observed in the distribution of sex and age group between included and excluded participants.

The sample plan considered 53 geographic domains (the 26 Brazilian states, the Federal District, and state capitals)^21^. The survey sample was selected by draw, based on a probabilistic sampling stratified by clusters (census tracts)^20,21^. Detailed information on planning can be found in the technical project of SB Brasil 2023^22^.

### Data collection

Data on tooth loss and sociodemographic variables (sex, self-reported ethnicity/race, per capita income, and level of education) were obtained from the SB Brasil 2023 database. Oral health coverage in PHC was obtained from the Health Information System for Primary Care (*Sistema de Informação em Saúde para a Atenção Básica* – SISAB), and the presence of CEO and LRPD was obtained from the National Registry of Health Establishments (*Cadastro Nacional de Estabelecimentos de Saúde* – CNES).

### Study variables

Tooth loss was defined as the main outcome. It was evaluated based on the result of the examination of Decayed, Missing, and Filled Teeth (DMFT), in relation to permanent teeth, of SB Brasil 2023, considering the component "M" (missing teeth), measured by clinical examination. Three dependent variables were analyzed:

1. Functional dentition (≥20 permanent teeth)^23^;
2. Severe tooth loss (<10 remaining permanent teeth)^23^; and
3. Edentulism (total loss of permanent teeth), based on the variable "edentulism" of the database.

The individual aspects from the epidemiological survey SB Brasil 2023 (sex, self-reported ethnicity/race, per capita income, and level of education) and contextual municipal aspects (oral health coverage in PHC, presence of CEO and LRPD) were considered independent variables. The predictive variables were categorized as:

- sex: women and men;
- self-reported ethnicity/race: Asian, white, Indigenous, mixed-race, and Black;
- Per capita household income: Up to the poverty line (BRL 665.00), poverty live up to one minimum wage (BRL 1,320.00), one to two minimum wages, and over two minimum wages;
- Level of education: Illiterate, one to four years of formal study, five to eight years of formal study, and over eight years of formal study;
- PHC coverage in the municipalities: <80% and ≥80%;
- Presence of Dental Specialty Centers (CEOs) in the municipalities: yes and no;
- Presence of Regional Dental Prosthesis Laboratories (LRPD) in the municipalities: yes and no.

### Statistical analyses

Descriptive and inferential statistical analyses were carried out. In the descriptive stage, the variables were presented in weighted absolute and relative frequencies, considering the sample weights and the complex design of SB Brasil 2023.

In the inferential analysis, odds ratio (OR) was estimated for the outcomes:

1. Functional dentition (≥20 permanent teeth);
2. Severe tooth loss (<10 permanent teeth); and
3. Edentulism (total absence of permanent teeth).

Multilevel logistic regression models were adjusted, with random intercept per municipality, to capture contextual variation.

The intraclass correlation coefficient (ICC) quantified the proportion of variability attributed to the municipal level in the null model. The adjustment was compared by using Akaike Information Criterion (AIC) and Bayesian Information Criterion (BIC) (lower values indicated better adjustment). The results were presented in OR and 95% confidence intervals (95%CI), with 5% significance (p<0.05). The analyses were carried out in Stata 19 (StataCorp LP, College Station, United States).

In addition, sensitivity analysis was performed by multilevel Poisson regression with robust variance in order to estimate prevalence ratios. According to the results, there was a pattern of associations consistent with the logistic models, with estimates of lower magnitude, as expected.

#### Data Availability Statement:

Data are available upon request. The derived database is available upon request to the corresponding author, while the original sources are official and accessible according to the rules of each system used for its development, present in the study methodology.

## RESULTS

Of the 9,745 older adults in SB Brasil 2023, 6,619 presented complete records and were included in the analysis. We observed a predominance of women, with per capita income between one and two minimum wages, and level of education between one and four years of formal study (Table 1).

**Table 1:** Descriptive characteristics and proportions. Study of the National Survey of Oral Health – SB Brasil 2023 (n=9,745).

Individual variables		Relative frequency (%) (weighted estimate)
Self-reported ethnicity/race		
	White	49.5 (45.8–53.3)
	Mixed-race	36.1 (32.4–40.2)
	Asian	1.1 (0.7–1.7)
	Black	12.8 (11.2–14.7)
	Indigenous	0.4 (0.2–1.0)
	Data not filled in	1.2 (0.7–2.0)
Sex		
	Women	60.4 (58.2–62.7)
	Men	39.6 (37.3–41.9)
Per capita income		
	Up to the poverty line	6.7 (5.3–8.4)
	Poverty line up to 1 minimum wage	27.6 (24.5–30.9)
	1 to 2 minimum wages	44.4 (40.7–48.3)
	Over 2 minimum wages	21.2 (18.0–24.9)
	Data not filled in	27.7 (23.4–32.5)
Level of education		
	Illiterate	11.4 (9.8–13.3)
	From 1 to 4 years	58.7 (55.4–61.9)
	From 5 to 8 years	20.7 (18.2–23.5)
	Over 8 years	9.2 (7.4–11.3)
	Data not filled in	3.8 (2.4–5.7)
Contextual variables		
Oral health coverage in Primary Health Care		
	Up to 70%	72.3 (67.0–77.0)
	>70%	27.7 (22.9–33.0)
Racial proportion		
	Up to 60% Black	65.8 (61.4–70.0)
	>60% Black	34.2 (30.0–38.6)
Dental Specialty Centers		
	No	26.9 (10.8–33.9)
	Yes	73.1 (66.1–79.2)
Prosthesis laboratories		
	No	24.2 (18.4–31.1)
	Yes	75.8 (68.9–81.6)
Functional dentition		
	No	80.4 (77.8–82.9)
	Yes	19.5 (17.2–22.2)
Severe loss		
	No	39.9 (36.6–43.4)
	Yes	60.1 (56.6–63.5)
Edentulism		
	No	63.5 (60.4–66.6)
	Yes	36.5 (33.4–39.6)

At the municipal level, about 70% of older adults lived in municipalities with access to oral health services in PHC and presence of CEO and LRPD. Most did not present edentulism, although there were high proportions of tooth loss and severe tooth loss (Table 1).

In the multilevel model for functional dentition (Table 2), income >2 minimum wages and higher level of education (5–8 and ≥9 years) were associated with higher odds of maintaining ≥20 teeth, while women and residents of municipalities with higher oral health coverage (≥80%) were associated with lower odds.

**Table 2:** Multilevel logistic regression that evaluated factors associated with the frequency of functional dentition (20 or more permanent teeth) in Brazilian older adults.

		Crude	Adjusted*
		OR (95%CI)	OR (95%CI)
Self-reported ethnicity/race (white)			
	Black	0.68 (0.55–0.84)	0.86 (0.70–1.05)
	Mixed-race	0.73 (0.63–0.85)	0.88 (0.76–1.02)
Sex (men)			
	Women	0.65 (0.57–0.74)	**0.66 (0.57–0.76)**
Level of education (illiterate)			
	1 to 4 years	1.32 (0.98–1.79)	1.3 (0.95–1.79)
	5 to 8 years	1.71 (1.28–2.29)	**1.6 (1.12–2.09)**
	≥9 years	3.95 (3.00–5.19)	**2.98 (2.11–4.20)**
Per capita household income (up to the poverty line)			
	Up to 1 minimum wage	0.90 (0.68–1.20)	0.9 (0.64–1.26)
	1 to 2 minimum wages	1.21(0.62–1.60)	1.01 (0.74–1.37)
	≥2 minimum wages	2.90 (2.18–3.86)	**1.78 (1.31–2.4)**
OH coverage in PHC (<80%)			
	≥80%	0.59 (0.49–0.74)	**0.74 (0.56–0.97)**
CEO presence (No)			
	Yes	1.68 (1.22–2.29)	1.21 (0.84–1.66)
LRPD presence (No)			
	Yes	1.52 (1.10–2.11)	1.17 (0.88–1.58)

OR: odds ratio; 95%CI: 95% confidence interval; CEO: Dental Specialty Centers; OH: Oral Health; PHC: Primary Health Care; LRPD: Regional Dental Prosthesis Laboratories.

Null model parameters: ICC [Intraclass Correlation Coefficient]=0.15; AIC [Akaike Information Criterion]=8521.6; BIC [Bayesian Information Criterion]=8535.9.

Adjusted model parameters: ICC=0.76; AIC=5640.4; BIC=5735.6.

Values in bold indicate statistically significant associations in the adjusted model.

For severe tooth loss (Table 3), we observed higher odds among women and older adults with income up to one minimum wage. Conversely, income >2 minimum wages, higher level of education (≥1 year), and residence in municipalities with CEO were associated with lower odds.

**Table 3:** Multilevel logistic regression that evaluated factors associated with the frequency of severe tooth loss (<10 remaining permanent teeth) in Brazilian older adults.

		Crude	Adjusted*
		OR (95%CI)	OR (95%CI)
Self-reported ethnicity/race (white)			
	Black	1.17 (0.99–1.37)	0.95 (0.81–1.12)
	Mixed-race	1.14 (1.01–1.29)	0.97 (0.86–1.10)
Sex (men)			
	Women	1.41(1.27–1.57)	**1.43 (1.27–1.62)**
Level of education (illiterate)			
	1 to 4 years	0.72 (0.59–0.88)	**0.70 (0.57–0.87)**
	5 to 8 years	0.55 (0.45–0.67)	**0.56 (0.45–0.71)**
	≥9 years	0.28 (0.23–0.33)	**0.33 (0.27–0.40)**
Per capita household income (up to the poverty line)			
	Up to 1 minimum wage	1.21 (0.99–1.48)	**1.27 (1.01–1.60)**
	1 to 2 minimum wages	1.01 (0.84–1.24)	1.22 (0.99–1.52)
	≥2 minimum wages	0.43 (0.35–0.54)	**0.67 (0.54–0.84)**
OH coverage in PHC (<80%)			
	≥80%	1.37 (1.07–1.55)	1.04 (0.80–1.31)
CEO presence (No)			
	Yes	0.60 (0.46–0.76)	**0.73 (0.56–0.95)**
LRPD presence (No)			
	Yes	0.69 (0.52–0.90)	0.91 (0.70–1.18)

OR: odds ratio; 95%CI: 95% confidence interval; CEO: Dental Specialty Centers; OH: Oral Health; PHC: Primary Health Care; LRPD: Regional Dental Prosthesis Laboratories.

Null model parameters: ICC=0.13; AIC=12559.9; BIC=12574.3.

Adjusted model parameters: ICC=0.76; AIC=8276.8; BIC=8371.9.

Values in bold indicate statistically significant associations in the adjusted model.

As for edentulism (Table 4), it was more frequent among women; income >2 minimum wages and higher level of education (1–4, 5–8, and ≥9 years) were protective factors. We found no association with municipal indicators of oral health services (PHC coverage, presence of CEO and LRPD).

**Table 4:** Multilevel logistic regression that evaluated factors associated with the frequency of edentulism (absence of all teeth) in Brazilian older adults.

		Crude	Adjusted*
		OR (95%CI)	OR (95%CI)
Self-reported ethnicity/race (white)			
	Black	1.03 (0.87–1.22)	0.84 (0.69–1.03)
	Mixed-race	1.07 (0.94–1.21)	0.91 (0.78–1.05)
Sex (men)			
	Women	1.32 (1.18–1.47)	**1.33 (1.19–1.49)**
Level of education (illiterate)			
	1 to 4 years	0.65 (0.55–0.78)	**0.64 (0.54–0.77)**
	5 to 8 years	0.51 (0.42–0.60)	**0.50 (0.42–0.60)**
	≥9 years	0.28 (0.24–0.33)	**0.30 (0.25–0.38)**
Per capita household income (up to the poverty line)			
	Up to 1 minimum wage	0.98 (0.80–1.19)	1.03 (0.83–1.26)
	1 to 2 minimum wages	0.82 (0.67–0.99)	0.97 (0.79–1.20)
	≥2 minimum wages	0.45 (0.36–0.56)	**0.69 (0.54–0.89)**
OH coverage in PHC (<80%)			
	≥80%	1.37 (1.09–1.71)	1.12 (0.89–1.40)
CEO presence (No)			
	Yes	0.68 (0.54–0.86)	0.86 (0.66–1.12)
LRPD presence (No)			
	Yes	0.67 (0.52–0.86)	**0.81 (0.63–1.05)**

OR: odds ratio; 95%CI: 95% confidence interval; CEO: Dental Specialty Centers; OH: Oral Health; PHC: Primary Health Care; LRPD: Regional Dental Prosthesis Laboratories.

Null model parameters: ICC=0.11; AIC=12376.5; BIC=12390.8.

Adjusted model parameters: ICC=0.75; AIC=7938.9; BIC=8034.1.

Values in bold indicate statistically significant associations in the adjusted model.

## DISCUSSION

According to the results, functional dentition and severe tooth loss in Brazilian older adults are associated with individual and contextual factors. For functional dentition, there was higher odds among older people with per capita household income ≥2 minimum wages and higher level of education, while women were associated with lower odds of dental preservation. At the municipal level, living in municipalities with higher oral health coverage in PHC (≥80%) was associated with lower odds of functional dentition, without evidence of association with the presence of CEO or LRPD. Edentulism was only associated with individual socioeconomic variables. As for severe tooth loss, on the one hand, the presence of CEO was associated with lower occurrence; on the other hand, the high coverage of OHT did not translate into better outcomes, suggesting the persistence of inequalities in access and effectiveness of care.

Regarding functional dentition, a slight improvement among Brazilian older adults was observed in recent decades: 10.8% in 2003, 13.6% in 2010, and 19.5% in the present study^4,24,25^. Despite the progress, inequalities remain. There was a lower prevalence of functional dentition among older adults residing in municipalities with higher oral health coverage in PHC, suggesting that the quantitative expansion of PHC teams does not always translate into better quality of care for this age group^26^.

This finding — lower odds of functional dentition in municipalities with higher OHT coverage — does not necessarily contradict the principles of PHC; it evidences that coverage may not reflect effective access, continuity, and problem-solving capacity, especially in historically vulnerable territories, where expansion may coexist with residual inequalities^26^. In addition, the results suggest that the interpretation should consider the care model: when a non-preventive pattern centered on late interventions predominates, greater supply can translate into more repeated procedures and low-preventive value, supporting the "restorative cycle" and contributing to cumulative loss of dental structure over time, without ensuring greater dental preservation^27–29^.

Even with PHC coordinating care services, there are limits to incorporate and sustain conservative strategies in complex cases, making integration with specialized care decisive to increase problem-solving capacity^30^. Procedures with greater potential for dental preservation depend on protocols and operating conditions — inputs, infrastructure, clinical time, and training — that are not always available in the PHC routine^31–33^. Thus, therapeutic decisions can favor faster solutions, such as dental extractions or referrals, evidencing that reducing tooth losses in older adults requires not only coverage, but problem-solving capacity and timely supply of conservative alternatives^30^.

The absence of the protective effect of OHT coverage on edentulism should be understood in the light of aging and the cumulative nature of this outcome: full edentulism results from exposures and decisions accumulated for decades. Hence, a contextual measure at a single point in time, such as the municipal coverage of OHT in 2023, is a static and little sensitive approach to represent the historical exposure to preventive and conservative care. This helps explain why contemporary associations may not translate into protection for already-established edentulism^34^. This set of elements helps to understand why socioeconomic inequalities remained strongly associated with outcomes in this population.

When analyzing individual factors, we observed that lower level of education and lower per capita household income are associated with lower prevalence of functional dentition. The concentration of tooth losses among socioeconomically-disadvantaged individuals^35–37^ — even considering variations in the municipal context described in the literature^37^ — remains a reality in Brazil, reflecting inequalities in the distribution and effectiveness of public oral health services.

Although PNSB guidelines have expanded the provision of services in the last decades^38,39^, the increase in the workforce does not seem to have been sufficient to consistently modify the pattern of exclusion in access to dental care^38^. Thus, in addition to expanding coverage, it is necessary to advance in the effectiveness, quality, and equity of care for the older adult population, especially in contexts of greater social vulnerability.

As for severe tooth loss, authors of a study conducted in 2019 estimated that about 59% of Brazilian older adults were affected^23^, and the findings of our research suggest persistence of this scenario, with approximately 60% older individuals affected. In addition, when compared to developed countries^41,42^, Brazil^40^ maintains higher averages of tooth loss in adults, reinforcing the influence of individual and contextual factors on the occurrence of this disease^43–45^.

From an etiological point of view, the magnitude and persistence of tooth loss may reflect the combined contribution of caries and periodontal disease to aging. Periodontal disease is a key determinant because it is a chronic inflammatory condition that, over time, promotes loss of insertion, mobility, and extractions, especially when diagnosis and support therapy occur late^46,47^. At the population level, severe periodontitis remains highly prevalent and concentrates great global burden, which helps explain important tooth losses in older adults, especially in contexts of greater vulnerability and lower access to preventive care and periodontal maintenance^48^. In this sense, the lowest severe tooth loss in municipalities with CEO is plausible by expanding access to specialized periodontal procedures and conservative approaches, potentially reducing avoidable extractions related to periodontal progression.

The lower prevalence of severe tooth loss in older adults residing in municipalities with CEO also converges with findings from the literature^49^, according to which there is an association between the presence of at least one CEO and the reduction of the proportions of extractions in the public network. This evidence reinforces the strategic role of these services in the qualification of care^50^, by enabling access to specialized and conservative procedures — periodontal, endodontic and rehabilitating — that contribute to preventing avoidable tooth losses.

From the point of view of public policies, oral health strategies should consider not only the implementation of services — such as CEO and LRPD —, but also their problem-solving capacity, quality of care, and equity in access. The physical presence of these services alone does not guarantee holistic, timely, and quality care^51,52^. Thus, systematic monitoring of performance indicators and continuity of care are necessary, in addition to equitable distribution of resources, to reduce inequalities in tooth losses and increase the impact of policies on the oral health of the population.

In our study, we identified that women had higher odds of edentulism. This result converges with national and global evidence showing higher prevalence of this outcome among women, especially in contexts of socioeconomic inequality^53,54^. Therefore, it reinforces the need to incorporate gender-specific approaches in public oral health policies, recognizing that social determinants of health are expressed differently between men and women and that strategies that are more equitable and sensitive to structural inequalities are essential to face edentulism in aging^55^.

The greater occurrence of tooth loss and edentulism among older women suggests a complex phenomenon, which extrapolates the scope of cross-sectional analyses and demands a deepening of life trajectories, access, and therapeutic decisions over time. This inequality may reflect the combination of greater female longevity, cumulative exposure to oral health issues, and specific social and economic barriers, in a context of accelerated and heterogeneous Brazilian aging. Likewise, the concept of Rapid Oral Health Deterioration (ROHD) describes the accelerated deterioration of oral health in older people after systemic and social decline, involving multimorbidity, polypharmacy, frailty, loss of autonomy, and difficulties in access to preventive and rehabilitative care^56^. Qualitative studies can clarify how gender, aging, and experience with services — including prioritization of extractions and barriers to rehabilitation — contribute to maintaining high levels of tooth loss among older women in Brazil, subsidizing more equitable and person-centered strategies.

The analysis of contextual variables did not consider the type of dental service used by the older adults, as the available question was only the place of the last visit, and the use of services in the last year was minimum. Furthermore, restricting the analysis to older adults whose last consultation was held by the SUS would excessively reduce the scope, considering the universality of the SUS. Nevertheless, we found no association between contextual indicators of oral health services and edentulism, possibly because it is a cumulative outcome and often established a long time ago. In addition, we could not incorporate the Municipal Human Development Index (MHDI) as a contextual variable, considering that there are no updated estimates compatible with the year 2023. Hence, we decided not to use lagged indicators — such as the 2010 MHDI — that could introduce classification error and obscure possible associations between the municipal socioeconomic context and tooth loss outcomes.

The collinearity between per capita household income and level of education constitutes a methodological limitation, as the high correlation between these variables makes it difficult to interpret the effects alone. We sought to mitigate this problem by using per capita income (household income divided by the number of residents) and level of education (individual attribute of the participant), which capture different dimensions. Nonetheless, we decided to keep them in the model because they represent complementary components of the social determinants of oral health and, together, expand the understanding of the analyzed outcomes.

As it is a cross-sectional survey, data were collected at a single point in time, which prevents causal inferences and only allows identifying associations. Thus, results — such as the association observed between the presence of CEO and lower tooth loss — should be interpreted with caution, as they may reflect both a compatible association with the benefit of the policy and a potential selection bias of more structured municipalities with better oral health conditions.

Moreover, multilevel logistic regression was used to incorporate the contextual level. However, we are aware that in cross-sectional studies with high prevalence outcomes, OR can overestimate the magnitude of the association in relation to the prevalence ratio (PR), usually estimated by Poisson regression with robust variance. Thus, this aspect should be considered in the interpretation, considering OR as association measures and not as direct PR approximations^57^.

Despite the limitations, our findings reinforce the existence of inequalities in tooth loss in Brazil and the need for longitudinal investigations that deepen the social and structural determinants of oral health in aging. We observed that sociodemographic and contextual factors are associated with different levels of tooth loss: higher income and level of education were associated with higher odds of functional dentition and lower odds of severe tooth loss and edentulism.

The presence of CEO in the municipality was a protective factor for severe tooth loss, suggesting a contribution of the provision of specialized services in the SUS. Conversely, women were consistently associated with worse outcomes, pointing to the need for intersectional approaches in public policies.

According to our results, extending the oral health coverage in PHC alone is not enough to improve dental conditions among older adults, and it is essential to evaluate the effectiveness and quality of services for more problem-solving responses. Thus, the findings subsidize the planning of public oral health services, guiding the allocation of resources, qualified expansion, and monitoring of social inequalities in oral health of the older adult population.
