# Supplementary material for: Individual and contextual factors associated with tooth loss among Brazilian older adults
Source: Rev Bras Epidemiol. 2026 May 18;29(Suppl 1):e260005supl1. doi: 10.1590/1980-549720260005.supl.1 (PMC13186543; doi:10.1590/1980-549720260005.supl.1)
Supplement: Translation

# Translation: Fatores individuais e contextuais associados à perda dentária em idosos brasileiros

**DOI:** 10.1590/1980-549720260005.supl.1.2

**Authors:** Armando Cabral de Lira, Ana Beatriz Rodrigues Moura, Lilian Nadja Silva Brito, Lucas Xavier Bezerra de Menezes, Luciana Leônia Soares Freire, Maria Letícia Barbosa Raymundo, Thayana Maria Navarro Ribeiro de Lima Martins, Rênnis Oliveira da Silva, Yuri Wanderley Cavalcanti, Rafael Aiello Bomfim

**Affiliations:** I Universidade Federal da Paraíba, Programa de Pós-Graduação em Odontologia – João Pessoa (PB), Brasil.; II Universidade Federal de Pernambuco, Programa de Pós-graduação em Saúde da Criança e do Adolescente – Recife (PE), Brasil.; III Universidade Federal da Paraíba, Departamento de Clínica e Odontologia Social – João Pessoa (PB), Brasil.; IV Universidade Federal do Mato Grosso do Sul, Departamento de Odontologia Preventiva – Campo Grande (MS), Brasil.

**Keywords:** Idoso, Perda dentária, Desigualdades em saúde, Saúde bucal, Fatores socioeconômicos, Estudos transversais

## Abstract

**Objetivo:**

Analisar fatores associados à dentição funcional, perda dentária severa e edentulismo em idosos brasileiros, considerando características sociodemográficas individuais e fatores contextuais dos serviços de saúde bucal.

**Métodos:**

Estudo transversal, quantitativo e analítico, baseado nos dados dos participantes de 65 a 74 anos da SB Brasil 2023. Analisou-se a perda dentária em três desfechos: dentição funcional (presença de pelo menos 20 dentes permanentes), perda severa (menos de dez dentes permanentes remanescentes) e edentulismo (perda de todos os dentes permanentes). Modelos de regressão logística multinível foram obtidos, considerando-se o delineamento amostral complexo. Variáveis individuais (sexo, etnia, renda familiar *per capita* e escolaridade) e contextuais (cobertura da saúde bucal na atenção básica, presença de Centros de Especialidades Odontológicas (CEO) e Laboratórios Regionais de Prótese Dentária [LRPD]) foram analisadas, considerando-se p<0,05.

**Resultados:**

A cobertura de saúde bucal na atenção básica e a presença de CEO e LRPD não foram associadas ao maior edentulismo (p>0,05). Observou-se menor prevalência de dentição funcional em mulheres (*odds ratio* — OR 0.66; intervalo de confiança de 95% — IC95% 0.57–0.76) e em residentes de municípios com cobertura de saúde bucal ≥80% (OR 0.74; IC95% 0.56–0.97). Idosos com maior renda e escolaridade tiveram maior prevalência de dentição funcional (p<0,05). Maior prevalência de perda dentária severa e edentulismo foi observada em mulheres e entre idosos com menor renda e menor escolaridade (p<0,05). Observou-se menor prevalência de perda severa em idosos residentes em municípios com CEO (p<0,05).

**Conclusão:**

Variáveis socioeconômicas individuais e características contextuais dos serviços de saúde bucal estiveram associadas à perda dentária em idosos brasileiros, evidenciando a persistência de desigualdades sociais nesse desfecho.

## INTRODUÇÃO

As doenças bucais estão entre as condições de saúde mais prevalentes no mundo e configuram importantes doenças crônicas não transmissíveis^1^. Entre seus desfechos, destaca-se a perda dentária, consequência cumulativa da cárie não tratada e da doença periodontal. Ao longo do curso da vida, essa carga pode resultar em perda dentária severa, definida pela Organização Mundial da Saúde (OMS) como a presença de menos de dez dentes permanentes^2^, com repercussões como dor e sofrimento, comprometimento funcional e redução da qualidade de vida^3^.

No Brasil, a Pesquisa Nacional de Saúde Bucal (SB Brasil 2010) mostrou médias de dentes perdidos de 0,4 em adolescentes (15–19 anos), 7,4 em adultos (35–44 anos) e 25,4 em idosos (65–74 anos)^4^. Essas diferenças etárias expressam efeitos cumulativos de um modelo histórico de atenção em saúde bucal marcado por práticas mutiladoras e por ações preventivas restritivas, insuficientes para responder às necessidades da população^5^.

Os idosos concentram o maior acúmulo de agravos bucais e, por isso, constituem um marcador sensível da efetividade das políticas públicas ao longo do tempo^6,7^. A perda severa compromete funções mastigatórias e aspectos estéticos, reduz a qualidade de vida e reflete desigualdades sociais, revelando a capacidade — ou insuficiência — do sistema público em oferecer cuidado preventivo e reabilitador de forma equitativa^8^. Ferreira et al.^9^ observaram reduções mais expressivas na perda dentária e no edentulismo entre idosos brancos e com maior escolaridade, mostrando que os avanços ocorreram de forma desigual entre grupos populacionais.

Nesse contexto, revisões sistemáticas e estudos de síntese internacionais mostram que a perda dentária em adultos e idosos é um problema global de saúde pública, com alta prevalência e fortes desigualdades sociais. Revisão sistemática de Chan et al.^10^ demonstrou que a cárie coronária e radicular segue altamente prevalente entre idosos em diferentes regiões, com médias elevadas de dentes perdidos e maior carga entre indivíduos socialmente vulneráveis e com acesso irregular aos serviços odontológicos. De forma complementar, consensos e revisões internacionais atestam que modelos assistenciais centrados em intervenções tardias, restauradoras ou cirúrgicas, são insuficientes para a complexidade do cuidado no envelhecimento, reforçando abordagens preventivas, de manejo longitudinal do risco e centradas na pessoa ao longo do curso da vida^11–13^.

Além das limitações atribuídas ao sistema público, a persistência do modelo mutilador também deve ser compreendida à luz do setor privado. A mercantilização da odontologia tende a reconfigurar o cuidado como serviço de consumo, com tratamentos ofertados como mercadorias, e não orientados pelo melhor interesse do paciente^14,15^. Nesse cenário, a extração pode se impor como alternativa rápida e de menor custo, sobretudo entre populações socialmente vulneráveis, reforçando práticas mutiladoras e desigualdades em saúde bucal. Evidências clínicas recentes demonstram que idade, restrições econômicas e acesso limitado a tratamentos conservadores associam-se a maior frequência de procedimentos cirúrgicos em detrimento de abordagens restauradoras, sugerindo que decisões terapêuticas são fortemente condicionadas por determinantes estruturais e organizacionais do cuidado^16^.

A Política Nacional de Saúde Bucal (PNSB), implementada em 2004 e instituída como política de Estado pela Lei n° 14.572/2023, busca ampliar a oferta de ações de promoção e prevenção e reduzir iniquidades no acesso à atenção odontológica^10,17^. A oferta de serviços ocorre por meio da Rede de Atenção à Saúde Bucal, que articula diferentes pontos de atenção. Na Atenção Primária à Saúde (APS), atuam as Equipes de Saúde Bucal (eSB) e as Unidades Odontológicas Móveis (UOM); na atenção especializada, destacam-se os Centros de Especialidades Odontológicas (CEO), o Serviço Especializado em Saúde Bucal (SESB) e os Laboratórios Regionais de Prótese Dentária (LRPD), compondo uma rede voltada à integralidade do cuidado^17^.

Apesar de formalmente estruturada, a rede tem sido marcada por subfinanciamento e subdimensionamento de recursos, gerando descompasso entre sua expansão e as necessidades da população. Como consequência, os serviços frequentemente oferecem respostas tardias, centradas no alívio da dor e em práticas predominantemente intervencionistas^18,19^. Nesse sentido, a formulação e implementação da PNSB reconheceu o papel dos determinantes sociais da saúde e priorizou a expansão da rede em contextos mais desfavorecidos, com piores indicadores de saúde^17^.

Nos últimos 20 anos, a população de 65 a 74 anos potencialmente se beneficiou da expansão da oferta de serviços públicos de saúde bucal no Sistema Único de Saúde (SUS). Diante desse cenário, é relevante investigar a associação de fatores socioeconômicos individuais e de características contextuais dos serviços de saúde bucal com a prevalência da perda dentária em idosos. Assim, o presente estudo tem como objetivo analisar os fatores individuais e contextuais associados à perda dentária em idosos brasileiros.

## MÉTODOS

### Delineamento do estudo

Trata-se de estudo transversal, quantitativo, de caráter descritivo e analítico, baseado nos dados do inquérito epidemiológico do SB Brasil 2023. A coleta ocorreu entre 2022 e 2023, seguindo diretrizes metodológicas padronizadas nacionalmente. O desenho amostral e os procedimentos metodológicos foram descritos por Vargas et al.^20^ e Ferreira et al.^9^.

O inquérito nacional foi aprovado por comitê de ética em pesquisa, e todos os participantes assinaram termo de consentimento livre e esclarecido. Os dados desta investigação foram disponibilizados pelo Ministério da Saúde, mediante solicitação e assinatura de termo de compromisso pelos pesquisadores.

### Amostra

A amostra foi composta de indivíduos idosos, com idade de 65–74 anos (n=9.745), que responderam ao questionário individual do SB Brasil 2023. Foi realizada análise de completude, e não foram observadas diferenças substantivas na distribuição de sexo e faixa etária entre incluídos e excluídos.

O plano amostral considerou 53 domínios geográficos (os 26 estados, Distrito Federal e capitais estaduais)^21^. A amostra do inquérito foi selecionada por sorteio, com base em uma amostragem probabilística estratificada por conglomerados (setores censitários)^20,21^. As informações detalhadas sobre o planejamento podem ser encontradas no projeto técnico do SB Brasil 2023^22^.

### Coleta de dados

Os dados de perda dentária e das variáveis sociodemográficas (sexo, declaração étnico-racial, renda *per capita* e escolaridade) foram obtidos do banco do SB Brasil 2023. A cobertura de saúde bucal na Atenção Básica (AB) foi extraída do Sistema de Informação em Saúde para a Atenção Básica (SISAB), e a presença de CEO e de LRPD foi obtida no Cadastro Nacional de Estabelecimentos de Saúde (CNES).

### Variáveis estudadas

Definiu-se a perda dentária como desfecho principal. Sua avaliação baseou-se no resultado do exame Cárie, Perdidos e Obturados em Dentes permanentes (CPO-D) do SB Brasil 2023, considerando-se o componente "P" (dentes perdidos), mensurado por exame clínico. Foram analisadas três variáveis dependentes:

1. Dentição funcional (≥20 dentes permanentes)^23^;
2. Perda dentária severa (<10 dentes permanentes remanescentes)^23^; e
3. Edentulismo (perda total de dentes permanentes), com base na variável "edentulismo" do banco de dados.

Foram consideradas variáveis independentes os aspectos individuais provenientes do inquérito epidemiológico do SB Brasil 2023 (sexo, declaração étnico-racial, renda *per capita* e escolaridade) e aspectos contextuais municipais (cobertura da atenção básica em saúde bucal, presença de CEO e presença de LRPD). As variáveis preditoras foram categorizadas como:

- sexo: feminino e masculino;
- declaração étnico-racial: amarelos, brancos, indígenas, pardos e pretos;
- renda familiar per capita: Até a linha da pobreza (R$ 665,00), pobreza até um salário-mínimo (R$ 1.320,00), um a dois salários-mínimos e acima de dois salários-mínimos;
- escolaridade: Não estudou, de um a quatro anos de estudo, de cinco a oito anos de estudo e acima de oito anos de estudo;
- cobertura da Atenção Básica em Saúde Bucal nos municípios: <80% e ≥80%;
- presença de Centros de Especialidades Odontológicas (CEO) nos municípios: sim e não;
- presença de Laboratórios Regionais de Próteses Dentárias (LRPD) nos municípios: sim e não.

### Análises estatísticas

Foram realizadas análises estatísticas descritivas e inferenciais. Na etapa descritiva, as variáveis foram apresentadas em frequências absolutas e relativas ponderadas, considerando-se os pesos amostrais e o delineamento complexo do SB Brasil 2023.

Na análise inferencial, estimaram-se razões de chances (*odds ratio* — OR) para os desfechos:

1. Dentição funcional (≥20 dentes permanentes);
2. Perda dentária severa (<10 dentes permanentes); e
3. Edentulismo (ausência total de dentes permanentes).

Ajustaram-se modelos de regressão logística multinível, com intercepto aleatório por município, para captar a variação contextual.

O coeficiente de correlação intraclasse (ICC) quantificou a proporção de variabilidade atribuída ao nível municipal no modelo nulo. O ajuste foi comparado por Critério de Informação de Akaike (AIC) e Critério de Informação Bayesiano (BIC) (menores valores indicaram melhor ajuste). Os resultados foram apresentados em OR e IC95%, com significância de 5% (p<0,05). As análises foram realizadas no Stata 19 (StataCorp LP, College Station, Estados Unidos).

Adicionalmente, realizou-se análise de sensibilidade por regressão de Poisson multinível com variância robusta, a fim de estimar razões de prevalência. Os resultados mostraram padrão de associações consistente com os modelos logísticos, com estimativas de menor magnitude, conforme esperado.

#### Declaração de Disponibilidade de Dados:

Dados disponíveis mediante solicitação. A base de dados derivada está disponível mediante solicitação ao autor correspondente, enquanto as fontes originais são oficiais e acessíveis conforme as regras de cada sistema utilizado para sua construção, presentes na metodologia do estudo.

## RESULTADOS

Dos 9.745 idosos do SB Brasil 2023, 6.619 apresentaram registros completos e foram incluídos na análise. Observou-se predominância do sexo feminino, com renda *per capita* entre um e dois salários-mínimos e escolaridade de um a quatro anos (Tabela 1).

**Tabela 1:** Características descritivas e proporções. Estudo da Pesquisa Nacional de Saúde Bucal — SB BRASIL 2023 (n=9745).

Variáveis individuais		Frequência relativa (%) (estimativa ponderada)
Declaração étnico-racial		
	Brancos	49,5 (45,8–53,3)
	Pardos	36,1 (32,4–40,2)
	Amarelo	1,1 (0,7–1,7)
	Pretos	12,8 (11,2–14,7)
	Indígenas	0,4 (0,2–1,0)
	Dados não preenchidos	1,2 (0,7–2,0)
Sexo		
	Feminino	60,4 (58,2–62,7)
	Masculino	39,6 (37,3–41,9)
Renda *per capita*		
	Até o nível de pobreza	6,7 (5,3–8,4)
	Pobreza até 1 salário-mínimo	27,6 (24,5–30,9)
	1 a 2 salários-mínimos	44,4 (40,7–48,3)
	Acima de 2 salários-mínimos	21,2 (18,0–24,9)
	Dados não preenchidos	27,7 (23,4–32,5)
Escolaridade		
	Analfabeto	11,4 (9,8–13,3)
	De 1 a 4 anos	58,7 (55,4–61,9)
	De 5 a 8 anos	20,7 (18,2–23,5)
	Acima de 8 anos	9,2 (7,4–11,3)
	Dados não preenchidos	3,8 (2,4–5,7)
Variáveis contextuais		
Cobertura saúde bucal na Atenção Básica		
	Até 70%	72,3 (67,0–77,0)
	>70%	27,7 (22,9–33,0)
Proporção racial		
	Até 60% pretos	65,8 (61,4–70,0)
	>60% pretos	34,2 (30,0–38,6)
Centros de Especialidades Odontológicas		
	Não	26,9 (10,8–33,9)
	Sim	73,1 (66,1–79,2)
Laboratórios de prótese		
	Não	24,2 (18,4–31,1)
	Sim	75,8 (68,9–81,6)
Dentição funcional		
	Não	80,4 (77,8–82,9)
	Sim	19,5 (17,2–22,2)
Perda severa		
	Não	39,9 (36,6–43,4)
	Sim	60,1 (56,6–63,5)
Edentulismo		
	Não	63,5 (60,4–66,6)
	Sim	36,5 (33,4–39,6)

No nível municipal, cerca de 70% dos idosos residiam em municípios com acesso a serviços de saúde bucal na Atenção Básica (AB) e presença de CEO e LRPD. A maioria não apresentava edentulismo, embora houvesse altas proporções de perda dentária e perda severa (Tabela 1).

No modelo multinível para dentição funcional (Tabela 2), renda >2 salários-mínimos e maior escolaridade (5–8 e ≥9 anos) associaram-se a maiores chances de manter ≥20 dentes, enquanto sexo feminino e residência em municípios com maior cobertura de saúde bucal (≥80%) associaram-se a menores chances.

**Tabela 2:** Regressão logística multinível que avaliou os fatores associados à frequência de dentição funcional (20 ou mais dentes permanentes) em idosos brasileiros.

		Não ajustado	Ajustado*
		OR (95%CI)	OR (95%CI)
Declaração étnico-racial (branca)			
	Preto	0,68 (0,55–0,84)	0,86 (0,70–1,05)
	Pardos	0,73 (0,63–0,85)	0,88 (0,76–1,02)
Sexo (Masculino)			
	Feminino	0,65 (0,57–0,74)	**0,66 (0,57–0,76)**
Escolaridade (não estudou)			
	1 a 4 anos	1,32 (0,98–1,79)	1,3 (0,95–1,79)
	5 a 8 anos	1,71 (1,28–2,29)	**1,6 (1,12–2,09)**
	≥9 anos	3,95 (3,00–5,19)	**2,98 (2,11–4,20)**
Renda familiar *per capita* (até a linha da pobreza)			
	Até 1 salário-mínimo	0,90 (0,68–1,20)	0,9 (0,64–1,26)
	1 a 2 salários-mínimos	1,21(0,62–1,60)	1,01 (0,74–1,37)
	≥ 2 salários-mínimos	2,90 (2,18–3,86)	**1,78 (1,31–2,4)**
Cobertura SB-AB (<80%)			
	≥80%	0,59 (0,49–0,74)	**0,74 (0,56–0,97)**
Presença de CEO (Não)			
	Sim	1,68 (1,22–2,29)	1,21 (0,84–1,66)
Presença de LRPD (Não)			
	Sim	1,52 (1,10–2,11)	1,17 (0,88–1,58)

OR: razão de chances (*odds ratio*); IC95%: intervalo de confiança 95%; CEO: Centros de Especialidades Odontológicas; SB: Saúde Bucal; AB: Atenção Básica; LRPD: Laboratórios Regionais de Prótese Dentária.

Parâmetros do modelo nulo: ICC=0,15; AIC=8521,6; BIC=8535,9.

Parâmetros do modelo ajustado: ICC=0,76; AIC=5640,4; BIC=5735,6.

Valores em negrito indicam associações estatisticamente significativas no modelo ajustado.

Para perda dentária severa (Tabela 3), observou-se maior chance entre mulheres e idosos com renda de até um salário-mínimo. Em contrapartida, renda >2 salários-mínimos, maior escolaridade (≥1 ano) e residência em municípios com CEO associaram-se a menores chances.

**Tabela 3:** Regressão logística multinível que avaliou os fatores associados à frequência de perda dentária severa (<10 dentes permanentes remanescentes) em idosos brasileiros.

		Não ajustado	Ajustado*
		OR (95%CI)	OR (95%CI)
Declaração étnico-racial (branca)			
	Preto	1,17 (0,99–1,37)	0,95 (0,81–1,12)
	Pardos	1,14 (1,01–1,29)	0,97 (0,86–1,10)
Sexo (Masculino)			
	Feminino	1,41(1,27–1,57)	**1,43 (1,27–1,62)**
Escolaridade (Não estudou)			
	1 a 4 anos	0,72 (0,59–0,88)	**0,70 (0,57–0,87)**
	5 a 8 anos	0,55 (0,45–0,67)	**0,56 (0,45–0,71)**
	≥9 anos	0,28 (0,23–0,33)	**0,33 (0,27–0,40)**
Renda familiar per capita (Até a linha da pobreza)			
	Até 1 salário-mínimo	1,21 (0,99–1,48)	**1,27 (1,01–1,60)**
	1 a 2 salários-mínimos	1,01 (0,84–1,24)	1,22 (0,99–1,52)
	≥2 salários-mínimos	0,43 (0,35–0,54)	**0,67 (0,54–0,84)**
Cobertura SB-AB (<80%)			
	≥80%	1,37 (1,07–1,55)	1,04 (0,80–1,31)
Presença de CEO (Não)			
	Sim	0,60 (0,46–0,76)	**0,73 (0,56–0,95)**
Presença de LRPD (Não)			
	Sim	0,69 (0,52–0,90)	0,91 (0,70–1,18)

OR: razão de chances (*odds ratio*); IC95%: intervalo de confiança 95%; CEO: Centros de Especialidades Odontológicas; SB: Saúde Bucal; AB: Atenção Básica; LRPD: Laboratórios Regionais de Prótese Dentária.

Parâmetros do modelo nulo: ICC=0,13; AIC=12559,9; BIC=12574.3.

Parâmetros do modelo ajustado: ICC=0,76; AIC=8276,8; BIC=8371,9.

Valores em negrito indicam associações estatisticamente significativas no modelo ajustado.

Quanto ao edentulismo (Tabela 4), foi mais frequente entre mulheres; renda >2 salários mínimos e maior escolaridade (1–4, 5–8 e ≥9 anos) atuaram como fatores protetores. Não houve associação com indicadores municipais dos serviços de saúde bucal (cobertura na AB, presença de CEO e LRPD).

**Tabela 4:** Regressão logística multinível que avaliou os fatores associados à frequência de edentulismo (ausência de todos os dentes) em idosos brasileiros.

		Não ajustado	Ajustado*
		OR (95%CI)	OR (95%CI)
Declaração étnico-racial (branca)			
	Preto	1,03 (0,87–1,22)	0,84 (0,69–1,03)
	Pardos	1,07 (0,94–1,21)	0,91 (0,78–1,05)
Sexo (Masculino)			
	Feminino	1,32 (1,18–1,47)	**1,33 (1,19–1,49)**
Escolaridade (não estudou)			
	1 a 4 anos	0,65 (0,55–0,78)	**0,64 (0,54–0,77)**
	5 a 8 anos	0,51 (0,42–0,60)	**0,50 (0,42–0,60)**
	≥9 anos	0,28 (0,24–0,33)	**0,30 (0,25–0,38)**
Renda familiar *per capita* (até a linha da pobreza)			
	Até 1 salário-mínimo	0,98 (0,80–1,19)	1,03 (0,83–1,26)
	1 a 2 salários-mínimos	0,82 (0,67–0,99)	0,97 (0,79–1,20)
	≥2 salários-mínimos	0,45 (0,36–0,56)	**0,69 (0,54–0,89)**
Cobertura SB-AB (<80%)			
	≥80%	1,37 (1,09–1,71)	1,12 (0,89–1,40)
Presença de CEO (Não)			
	Sim	0,68 (0,54–0,86)	0,86 (0,66–1,12)
Presença de LRPD (Não)			
	Sim	0,67 (0,52–0,86)	**0,81 (0,63–1,05)**

OR: razão de chances (*odds ratio*); IC95%: intervalo de confiança 95%; CEO: Centros de Especialidades Odontológicas; SB: Saúde Bucal; AB: Atenção Básica; LRPD: Laboratórios Regionais de Prótese Dentária.

Parâmetros do modelo nulo: ICC=0,11; AIC=12376,5; BIC=12390,8.

Parâmetros do modelo ajustado: ICC=0,75; AIC=7938,9; BIC=8034,1.

Valores em negrito indicam associações estatisticamente significativas no modelo ajustado.

## DISCUSSÃO

Os resultados demonstram que dentição funcional e perda dentária severa em idosos brasileiros associam-se a fatores individuais e contextuais. Para dentição funcional, observou-se maior chance entre idosos com renda familiar *per capita* ≥2 salários-mínimos e maior escolaridade, enquanto o sexo feminino associou-se a menor chance de preservação dentária. No nível municipal, residir em municípios com maior cobertura de saúde bucal na Atenção Básica (≥80%) associou-se a menor chance de dentição funcional, sem evidência de associação com a presença de CEO ou LRPD. O edentulismo associou-se apenas às variáveis socioeconômicas individuais. Quanto à perda dentária severa, a presença de CEO associou-se a menor ocorrência; em contrapartida, a alta cobertura de eSB não se traduziu em melhores desfechos, sugerindo a persistência de desigualdades no acesso e na efetividade do cuidado.

No que se refere à dentição funcional, observou-se discreta melhora entre idosos brasileiros nas últimas décadas: 10,8% em 2003, 13,6% em 2010 e 19,5% na presente pesquisa^4,24,25^. Apesar do avanço, as desigualdades permanecem. Registrou-se menor prevalência de dentição funcional entre idosos residentes em municípios com maior cobertura de saúde bucal na Atenção Básica, sugerindo que a expansão quantitativa das equipes na APS nem sempre se traduz em melhor qualidade do cuidado para essa faixa etária^26^.

Este achado — menor chance de dentição funcional em municípios com maior cobertura de eSB — não contradiz, necessariamente, os princípios da APS; mostra que cobertura pode não refletir acesso efetivo, longitudinalidade e resolutividade, sobretudo em territórios historicamente vulneráveis, onde a expansão pode conviver com desigualdades residuais^26^. Além disso, os resultados sugerem que a interpretação deve considerar o modelo de atenção: quando predomina um padrão pouco preventivo e centrado em intervenções tardias, maior oferta pode se traduzir em mais procedimentos repetidos e baixo valor preventivo, sustentando o "ciclo restaurador" e contribuindo para perda cumulativa de estrutura dentária ao longo do tempo, sem assegurar maior preservação dentária^27–29^.

Mesmo com a APS coordenando o cuidado, há limites para incorporar e sustentar estratégias conservadoras em casos complexos, tornando a integração com a atenção especializada decisiva para ampliar a resolutividade^30^. Procedimentos com maior potencial de preservação dentária dependem de protocolos e condições operacionais — insumos, infraestrutura, tempo clínico e capacitação — que nem sempre estão disponíveis na rotina da atenção primária^31–33^. Assim, decisões terapêuticas podem favorecer soluções mais rápidas, como exodontias ou encaminhamentos, mostrando que reduzir perdas dentárias em idosos requer não apenas cobertura, mas capacidade resolutiva e oferta oportuna de alternativas conservadoras^30^.

A ausência de efeito protetor da cobertura de eSB sobre o edentulismo deve ser compreendida à luz do envelhecimento e do caráter cumulativo desse desfecho: a perda total de dentes resulta de exposições e decisões acumuladas por décadas. Assim, uma medida contextual em um único ponto no tempo, como a cobertura municipal de eSB em 2023, é uma aproximação estática e pouco sensível para representar a exposição histórica ao cuidado preventivo e conservador, o que ajuda a explicar por que associações contemporâneas podem não se traduzir em proteção para edentulismo já instalado^34^. Esse conjunto de elementos contribui para entender por que as desigualdades socioeconômicas permaneceram fortemente associadas aos desfechos nesta população.

Ao analisar os fatores individuais, observou-se que menor escolaridade e menor renda familiar *per capita* se associam a menor prevalência de dentição funcional. A concentração de perdas dentárias entre indivíduos socioeconomicamente menos favorecidos^35–37^ — mesmo considerando variações do contexto municipal descritas na literatura^37^ — permanece uma realidade no Brasil, refletindo desigualdades na distribuição e na efetividade dos serviços públicos de saúde bucal.

Embora as diretrizes da PNSB tenham ampliado a oferta de serviços nas últimas décadas^38,39^, o aumento da força de trabalho não parece ter sido suficiente para modificar de forma consistente o padrão de exclusão no acesso aos cuidados odontológicos^38^. Assim, além de expandir cobertura, é necessário avançar na efetividade, na qualidade e na equidade do cuidado à população idosa, especialmente em contextos de maior vulnerabilidade social.

Quanto à perda dentária severa, estudo de 2019 estimou que cerca de 59% dos idosos brasileiros eram afetados^23^, e os achados desta pesquisa sugerem persistência desse cenário, com aproximadamente 60% acometidos. Além disso, o Brasil^40^, em comparação a países desenvolvidos^41,42^, mantém médias mais elevadas de perda dentária em adultos, reforçando a influência de fatores individuais e contextuais na ocorrência desse agravo^43–45^.

Do ponto de vista etiológico, a magnitude e a persistência da perda dentária podem refletir a contribuição combinada de cárie e doença periodontal no envelhecimento. A doença periodontal é determinante central por ser uma condição inflamatória crônica que, ao longo do tempo, promove perda de inserção, mobilidade e extrações, sobretudo quando diagnóstico e terapia de suporte ocorrem tardiamente^46,47^. Em nível populacional, a periodontite severa permanece altamente prevalente e concentra grande carga global, o que ajuda a explicar perdas dentárias importantes em adultos mais velhos, especialmente em contextos de maior vulnerabilidade e menor acesso a cuidado preventivo e manutenção periodontal^48^. Nesse sentido, a menor perda dentária severa em municípios com CEO é plausível pela ampliação do acesso a procedimentos periodontais especializados e a abordagens conservadoras, potencialmente reduzindo extrações evitáveis relacionadas à progressão periodontal.

A menor prevalência de perda dentária severa em idosos residentes em municípios com CEO também converge com achados da literatura^49^, que apontam associação entre a presença de pelo menos um CEO e a redução das proporções de exodontias na rede pública. Essa evidência reforça o papel estratégico desses serviços na qualificação do cuidado^50^, ao viabilizarem o acesso a procedimentos especializados e conservadores — periodontais, endodônticos e reabilitadores — que contribuem para prevenir perdas dentárias evitáveis.

Do ponto de vista das políticas públicas, as estratégias em saúde bucal devem considerar não apenas a implantação de serviços — como CEO e LRPD —, mas sua capacidade resolutiva, qualidade da atenção e equidade no acesso. A presença física desses serviços, por si só, não garante cuidado integral, oportuno e de qualidade^51,52^. Assim, são necessários o monitoramento sistemático de indicadores de desempenho e a continuidade da atenção, além da distribuição equitativa de recursos, para reduzir desigualdades nas perdas dentárias e ampliar o impacto das políticas na saúde bucal da população.

A presente pesquisa identificou que pessoas do sexo feminino apresentaram maior chance de edentulismo. Esse resultado converge com evidências nacionais e globais que mostram maior prevalência desse desfecho entre mulheres, especialmente em contextos de desigualdade socioeconômica^53,54^. Assim, reforça-se a necessidade de incorporar recortes de gênero nas políticas públicas de saúde bucal, reconhecendo que os determinantes sociais da saúde se expressam de forma distinta entre homens e mulheres e que estratégias mais equitativas e sensíveis às desigualdades estruturais são essenciais para enfrentar o edentulismo no envelhecimento^55^.

A maior ocorrência de perda dentária e edentulismo entre mulheres idosas sugere um fenômeno complexo, que extrapola o escopo de análises transversais e demanda aprofundamento sobre trajetórias de vida, acesso e decisões terapêuticas ao longo do tempo. Essa desigualdade pode refletir a combinação de maior longevidade feminina, exposição cumulativa a agravos bucais e barreiras sociais e econômicas específicas, em um contexto de envelhecimento brasileiro acelerado e heterogêneo. Nessa direção, o conceito de Rapid Oral Health Deterioration (ROHD) descreve a deterioração acelerada da saúde bucal em idosos após declínio sistêmico e social, envolvendo multimorbidade, polifarmácia, fragilidade, perda de autonomia e dificuldades de acesso a cuidado preventivo e reabilitador^56^. Estudos qualitativos podem esclarecer como gênero, envelhecimento e experiências com serviços — incluindo priorização de extrações e barreiras à reabilitação — contribuem para a manutenção de altos níveis de perda dentária entre mulheres idosas no Brasil, subsidiando estratégias mais equitativas e centradas na pessoa.

A análise das variáveis contextuais não considerou o tipo de serviço odontológico utilizado pelos idosos, pois a pergunta disponível referia-se apenas ao local da última consulta, e a utilização de serviços no último ano foi baixa. Além disso, restringir a análise aos idosos cuja última consulta ocorreu no SUS reduziria excessivamente o escopo, considerando-se a universalidade do SUS. Ainda assim, não se observou associação entre os indicadores contextuais dos serviços de saúde bucal e o edentulismo, possivelmente por se tratar de um desfecho cumulativo e frequentemente instalado há muito tempo. Adicionalmente, não foi possível incorporar o Índice de Desenvolvimento Humano Municipal (IDHM) como variável contextual, uma vez que não há estimativas atualizadas compatíveis com o ano de 2023; assim, optou-se por não utilizar indicadores defasados — como o IDHM de 2010 — que poderiam introduzir erro de classificação e obscurecer possíveis associações entre o contexto socioeconômico municipal e os desfechos de perda dentária.

A colinearidade entre renda familiar *per capita* e escolaridade constitui limitação metodológica, pois a alta correlação entre essas variáveis dificulta a interpretação isolada de seus efeitos. Buscou-se mitigar esse problema ao utilizar renda *per capita* (renda familiar dividida pelo número de moradores) e escolaridade (atributo individual do participante), que captam dimensões distintas. Ainda assim, optou-se por mantê-las no modelo por representarem componentes complementares dos determinantes sociais da saúde bucal e, em conjunto, ampliarem a compreensão dos desfechos analisados.

Por se tratar de uma pesquisa transversal, os dados foram coletados em um único ponto no tempo, o que impede inferências causais e permite apenas identificar associações. Assim, resultados como a associação observada entre a presença de CEO e a menor perda dentária devem ser interpretados com cautela, pois podem refletir tanto uma associação compatível com benefício da política quanto um potencial viés de seleção de municípios mais estruturados e com melhores condições de saúde bucal.

Adicionalmente, utilizou-se regressão logística multinível para incorporar o nível contextual. Reconhece-se, porém, que em estudos seccionais com desfechos de alta prevalência a OR pode superestimar a magnitude da associação em relação à razão de prevalência (RP), usualmente estimada por regressão de Poisson com variância robusta. Assim, esse aspecto deve ser considerado na interpretação, tratando as OR como medidas de associação e não como aproximações diretas de RP^57^.

Apesar das limitações, os achados reforçam a existência de desigualdades na perda dentária no Brasil e a necessidade de investigações longitudinais que aprofundem os determinantes sociais e estruturais da saúde bucal no envelhecimento. Observou-se que fatores sociodemográficos e contextuais se associam a diferentes níveis de perda dentária: maior renda e escolaridade associaram-se a maiores chances de dentição funcional e a menores chances de perda dentária severa e edentulismo.

A presença de CEO no município mostrou-se fator protetor para perda dentária severa, sugerindo contribuição da oferta de serviços especializados no SUS. Em contrapartida, o sexo feminino associou-se consistentemente a piores desfechos, apontando a necessidade de abordagens interseccionais nas políticas públicas.

Os resultados também mostram que ampliar apenas a cobertura da Atenção Básica em saúde bucal não basta para melhorar condições dentárias em idosos, sendo essencial avaliar efetividade e qualidade dos serviços para respostas mais resolutivas. Assim, os achados subsidiam o planejamento dos serviços públicos de saúde bucal, orientando a alocação de recursos, a expansão qualificada e o monitoramento das desigualdades sociais na saúde bucal da população idosa.
